# TMAO-Activated Hepatocyte-Derived Exosomes Impair Angiogenesis *via* Repressing CXCR4

**DOI:** 10.3389/fcell.2021.804049

**Published:** 2022-01-31

**Authors:** Xiang Liu, Yijia Shao, Jiazichao Tu, Jiapan Sun, Bing Dong, Zhichao Wang, Jianrong Zhou, Long Chen, Jun Tao, Jimei Chen

**Affiliations:** ^1^ Department of Cardiac Surgery, Guangdong Cardiovascular Institute, Guangdong Provincial People’s Hospital, Guangdong Academy of Medical Sciences, Guangzhou, China; ^2^ Guangdong Provincial Key Laboratory of South China Structural Heart Disease, Guangzhou, China; ^3^ School of Medicine, South China University of Technology, Guangzhou, China; ^4^ Department of Hypertension and Vascular Diseases, The First Affiliated Hospital, Sun Yat-sen University, Guangzhou, China; ^5^ NHC Key Laboratory of Assisted Circulation, Sun Yat-sen University, Guangzhou, China; ^6^ Department of Geriatrics, Peking University Shenzhen Hospital, Shenzhen Peking University-The Hong Kong University of Science and Technology Medical Center, Shenzhen, China; ^7^ The International Medical Department of Shenzhen Hospital, Southern Medical University, Shenzhen, China

**Keywords:** trimethylamine-N-oxide, hepatocyte-derived exosomes, endothelial function, angiogenesis, C-X-C motif chemokine receptor 4, alternative polyadenylation

## Abstract

**Objective:** Trimethylamine-N-oxide (TMAO) was found to play crucial roles in vascular endothelial function. However, the exact molecular mechanisms are not yet entirely clear. Recently, we found that exosomes (Exos) isolated from TMAO-treated hepatocytes (TMAO-Exos) contained a distinctive profile of miRNAs compared to those from the TMAO-free group (Control-Exos). Furthermore, TMAO-Exos could notably promote inflammation, damage vascular endothelial cells (VECs), and impair endothelium-dependent vasodilation. This study aimed to further evaluate the effects of TMAO-Exos on VECs and explore the underlying mechanisms.

**Methods:** Exos were isolated from the hepatocyte culture supernatant with or without TMAO, using differential centrifugation. Then, VECs were treated with these Exos for 48 h and subjected to RNA-sequencing for detecting the changes of alternative polyadenylation (APA) and mRNA. After validation by qPCR and western blotting, the recombinant viruses were used to mediate the overexpression of C-X-C motif chemokine receptor 4 (CXCR4). The *in vitro* VEC function was evaluated by cell migration and tube formation, and *in vivo* angiogenesis was investigated in hindlimb ischemia models.

**Results:** Exos released from hepatocytes were differentially regulated by TMAO; both could be taken up by VECs; and furthermore, TMAO-Exos significantly reduced cell migration and tube formation *in vitro* and impaired perfusion recovery and angiogenesis after hindlimb ischemia, by down-regulating the CXCR4 expression. However, TMAO-Exos failed to regulate the splicing events, at least in this experimental setting, which suggested that TMAO-Exos may affect CXCR4 expression *via* an APA-independent manner.

**Conclusions:** Our findings revealed a novel indirect mechanism by which TMAO impaired endothelial function through stimulating hepatocytes to produce Exos that possessed distinctive activity. The crosstalk between the liver and vascular endothelial mediated by these Exos may offer a new target for restraining the harmful effects induced by TMAO.

## Introduction

Gut microbiota has received increasing attention for the crucial roles in cardiovascular disease (Brown and Hazen, 2018). Trimethylamine-N-oxide (TMAO), known as one of the most important metabolites of intestinal flora, has been found to be an independent risk factor for adverse cardiovascular events (Wang et al., 2011; Tang et al., 2013; Tang et al., 2014; Zhu et al., 2016; Li X. S. et al., 2017). Furthermore, TMAO has been shown to induce inflammation and impair endothelial function including angiogenesis (Seldin et al., 2016; Li T. et al., 2017; Ke et al., 2018; Singh et al., 2019; Brunt et al., 2020; Chen et al., 2020; Liu and Dai, 2020). However, there is still a lot more down there that has to be uncovered.

Exosomes (Exos) are small membrane vesicles secreted by most cells, ranging from 50 to 100 nm in diameter. Exos have not only been assumed to be a biomarker and prognosticator in certain disease states but have also been found to play vital function in intercellular communication (Kourembanas, 2015; Ibrahim and Marban, 2016; Vidal, 2019). Recently, Exos derived from hepatocytes were shown to play an important role in inflammation, vascular endothelial function, and metabolic disorders (Momen-Heravi et al., 2015; Hirsova et al., 2016; Jiang et al., 2020; Ji et al., 2021; Luo et al., 2021; Nakao et al., 2021). In our latest work, Exos isolated from hepatocytes stimulated with TMAO (TMAO-Exos) contained a distinctive profile of miRNAs compared to those from the TMAO-free group (Control-Exos). Moreover, TMAO-Exos, but not Control-Exos, could significantly promote inflammation, damage vascular endothelial cells (VECs), and impair endothelium-dependent vasodilation (Liu et al., 2021b).

Alternative polyadenylation (APA) is an important post-transcriptional regulatory mechanism, leading to protein diversity by regulating the length of the 3ʹ-untranslated region (3ʹ-UTR) of each gene in the genome. It has been proved that shortening or lengthening of the 3ʹ-UTR can affect mRNA stability, translation efficiency, and protein localization (Gruber and Zavolan, 2019). Recent research revealed that APA played an important role in vascular endothelial function, such as angiogenesis, cell proliferation, migration, and vascular inflammation (Kern et al., 2008; Murphy and Hynes, 2014; Murphy et al., 2018; Bowler and Oltean, 2019). However, it is unclear whether TMAO-Exos can affect endothelial function *via* an APA-dependent manner.

In the study, to further explore the mechanisms by which TMAO-Exos impair endothelial function, high-throughput sequencing was performed to identify the transcriptome-wide profiling of alternative splicing and gene expression. The results showed that C-X-C motif chemokine receptor 4 (CXCR4) was notably decreased by TMAO-Exos, independent of alternative splicing, thus leading to reduced cell migration and tube formation and impaired perfusion recovery after hindlimb ischemia, which could be rescued by overexpression of CXCR4. It is hoped that this study will provide a light on the mechanisms behind TMAO, from the perspective of the crosstalk between the liver and vascular endothelial mediated by Exos.

### Materials and Methods

### Cell Culture, Exosome Isolation, Identification, and Labelling

AML12 cells were closely phenocopied by primary mouse hepatocytes and therefore have been widely used (Wu et al., 1994; Chen et al., 2019; Nagarajan et al., 2019). AML12 cells (iCell Bioscience Inc, Shanghai, China) were cultured in Dulbecco’s Modified Eagle Medium/Nutrient Mixture F-12 (DMEM/F12) containing Exos-depleted serum (ViVaCell, Shanghai, China), and treated with TMAO (Tokyo Chemical Industry, Japan) at a physiological concentration of 50 μmol/l for 48 h. Exos were isolated and purified from the culture supernatant using differential centrifugation and then resuspended in phosphate-buffered saline (PBS), as described in our previous study (Liu et al., 2021b). The protein levels of the Exos were measured using bicinchoninic acid (BCA) protein assay kit (Thermo Fisher Scientific, MA, United States). The concentration and size distribution of the Exos were detected by nanoparticle tracking analysis (Nanosight NS300, Malvern, UK). The ultrastructure of the Exos was inspected using a transmission electron microscopy (JEM1200-EX, Japan). Briefly, Exos suspensions were loaded on 200-mesh formvar-coated grids and then negatively stained with phosphotungstic acid. The samples were observed under a transmission electron microscope at a voltage of 100 kV. Exosomal markers of CD9 and TSG101 and negative marker of calnexin were detected by western blot. Exos were labelled with DiI (Beyotime Biotechnology, Shanghai, China) for *in vitro* and *in vivo* tracer experiments.

### Detection of Trimethylamine-N-Oxide

TMAO contained in the Exos was detected on a liquid chromatography-tandem mass spectrometry (LC-MS/MS) system consisting of an Agilent 1260 high-performance liquid chromatography (HPLC) and 6420 triple quadrupole mass spectrometer with an electrospray ionization source (ESI) (Agilent), which was performed according to the standard protocols as described in our previous study (Liu et al., 2021a). Briefly, 100 μl of the specimen was mixed with the internal standard working solution (d9-TMAO), and 300 μl of acetonitrile was added to the sample and then vortexed, and the mixture was centrifuged at 15,294×*g* for 5 min. Finally, the supernatant was transferred into an autosampler vial, and 2 μl was injected into the HPLC-MS/MS for analysis. Chromatographic separation was performed on the Waters Atlantis HILIC Silica column (3.0 × 100 mm, 3.0 μm), and the ESI was operated in positive mode, and the mass spectrometer was run in multiple-reaction monitoring mode.

### Cell Culture and Treatment

Primary human aortic endothelial cells (HAECs, iCell Bioscience Inc, Shanghai, China) were cultured in endothelial cell medium (ECM, ScienCell, CA, United States) supplemented with 5% fetal bovine serum, 1% growth factors, and 1% penicillin/streptomycin. Cells were treated with Exos at a concentration of 10 μg/ml. Lentivirus vectors carrying CXCR4 gene (LV-CXCR4) were constructed (Hanbio, Shanghai, China) to up-regulate the expression of CXCR4 *in vitro*. LV-CXCR4 was added to HAECs at a multiplicity of infection of 30, and empty vector served as negative control (LV-NC). The culture medium was replaced after 4 h and then treated with Exos for another 48 h.

### RNA-Sequencing and Bioinformatics Analysis

APA sequencing was performed as described previously (Fu et al., 2015; Fu et al., 2018). Total RNA was isolated using TRIzol reagent (Invitrogen, United States), and genome DNA was removed by Ambion Turbo DNA-free Kit (Invitrogen, United States), and then total RNA was randomly fragmented by heating. The first round of reverse transcription was performed using SuperScript III Kit (Thermo Fisher Scientific, MA, United States), and double-strand DNA was extracted with Agencourt RNAClean XP Kit (Beckman, CA, United States). The *in vitro* transcription RNA synthesis and purification were performed using RiboMAX^TM^ Large Scale RNA Production Systems (Promega, WI, United States), and then the second round of reverse transcription and double-strand DNA purification were performed. After PCR amplification, fragments between 300 and 500 bp were selected with Agencourt AMPure XP beads (Beckman, CA, United States), and the library preparations were sequenced on an Illumina NovaSeq 6000 platform.

Sequencing data were analyzed with in-house bioinformatics pipeline (Fu et al., 2011; Fu et al., 2015). Briefly, the raw reads were mapped to the human genome (hg19), and internal priming was filtered. Poly (A) sites were defined for each sample by clustering the unique mapped reads and then merged together across samples. Next, 3′-UTR length was standardized by designating the longest 3′-UTR as 1.0 and calculating the weighted mean length with multiple APA sites for each gene, and tandem 3′-UTR length difference between samples was detected by a test of linear trend alternative to independence. The Benjamin–Hochberg FDR was obtained, and FDR < 0.01 and |*r*| > 0.1 were set as the significant threshold.

The differentially expressed genes were screened according to the criteria of fold change >1.5 and *p* < 0.05. The candidate genes were visualized on a heatmap constructed in R. Database for Annotation, Visualization, and Integrated Discovery (DAVID) was used for functional annotation. Gene Ontology (GO) analysis was performed to elaborate the biological process, molecular function, and cellular component. Kyoto Encyclopedia of Genes and Genomes (KEGG) pathway enrichment was used to explore the relevant signal pathway. STRING database (v11.0) (Szklarczyk et al., 2019) was used for analyzing the protein–protein interaction (PPI), and networks were performed on Cytoscape platform (v3.8.2) (Shannon et al., 2003). CytoHubba plug-in was used to identify the hub genes with a threshold value >0.4, and the color of the nodes represents the degree of gene interaction.

### Western Blot Analysis

Western blot was performed according to the procedures as previously described (Zhou et al., 2018; Liu et al., 2021b). The protein concentration was determined using BCA Protein Assay Kit (Thermo Fisher Scientific, MA, United States). Then, the samples were separated by SDS-PAGE and transferred onto Millipore polyvinylidene difluoride membranes. The membranes were blocked with 5% bovine serum albumin for 1 h at room temperature and incubated overnight at 4°C with the primary antibodies of CD9 (ZEN BIO, Chengdu, China), TSG101 (ZEN BIO, Chengdu, China), calnexin (Affinity Biosciences, Jiangsu, China), CXCR4 (Abcam, MA, United States), β-tubulin (Ray Antibody Biotech, Beijing, China), and GAPDH (Proteintech Group, IL, United States). Then, the membranes were incubated with anti-rabbit or anti-mouse IgG-HRP (Santa Cruz Biotechnology, CA, United States) for 1 h at room temperature and visualized with enhanced chemiluminescence reagent (Millipore, MA, United States).

### Quantitative Polymerase Chain Reaction

Quantitative polymerase chain reaction (qPCR) was performed as described in our previous study (Zhou et al., 2018). Briefly, total RNA was extracted using TRIzol reagent (Invitrogen, United States), and concentration was measured using a NanoDrop 2000 spectrophotometer (Thermo Fisher Scientific, MA, United States). Then, RNA was reversely transcribed into cDNA using Color Reverse Transcription Kit (EZBioscience, CA, United States), and qPCR was performed on Bio-Rad CFX-96 (Bio-Rad, CA, United States) with Color SYBR Green qPCR Master Mix (EZBioscience, CA, United States). The CXCR4 levels were normalized to GAPDH. The qPCR primers used in the study are listed in Table 1.

**TABLE 1:** qPCR primers for mRNA used in the study.

Species	Name	Forward sequence	Reverse sequence
*Homo sapiens*	CXCR4	TCT​TCC​TGC​CCA​CCA​TCT​ACT​C	GTA​GAT​GAC​ATG​GAC​TGC​CTT​GC
*Homo sapiens*	GAPDH	TGC​ACC​ACC​AAC​TGC​TTA​GC	GGC​ATG​GAC​TGT​GGT​CAT​GAG
*Mus musculus*	CXCR4	CGT​CAT​CCT​CTC​CTG​TTA​CTG​C	GTC​GAT​GCT​GAT​CCC​CAC​ATA​A
*Mus musculus*	GAPDH	ACT​CTT​CCA​CCT​TCG​ATG​CC	TGG​GAT​AGG​GCC​TCT​CTT​GC

CXCR4, C-X-C motif chemokine receptor 4; GAPDH, glyceraldehyde phosphate dehydrogenase.

### Cell Scratch Assay

The procedure was performed as previously described (Ou et al., 2016). Briefly, scraping the cell monolayer in a straight line to create a “scratch” with a sterile p200 pipette. Debris was removed gently by washing with PBS. Then, the cells continued to be incubated with Exos, and images at 0 and 12 h were captured using inversion microscope.

### Tube Formation

The tube formation assay was performed by using basement membrane matrix gel as previously described (Ou et al., 2016). Briefly, Matrigel was thawed at 4°C and mixed with an equal part of ECM, then the mixture was added to the 96-well culture plates. After polymerization, HAECs with a density of 3 × 10^4^ were seeded to the Matrigel-coated plates and grown for 6 h. Then, the extent of tube formation was photographed by a microscope.

### Animal Experiments

All animal experiments have been approved by the committee review of animal experiments in Guangdong Provincial People’s Hospital (No. KY-D-2021-438-01). Eight-week-old male wild-type C57BL/6 mice were purchased from the Experimental Animal Center of Sun Yat-sen University and kept under controlled environmental conditions (constant laminar airflow, 20°C –23°C, 40%–60% relative humidity, and 12/12-h light/dark cycle). To evaluate the *in vivo* distribution of Exos in aortas and the effects of Exos on CXCR4, mice were intravenously injected with 30 μg of Exos or Dil-Exos resuspended in 100 μl of PBS. After 24 h, the mice were anesthetized by pentobarbital sodium (50 mg/ kg), and aortas were collected for subsequent study.

The hindlimb ischemia models were performed as described in our previous study (Yu et al., 2019). The adeno-associated virus (AAV) vectors encoding CXCR4 (AAV-CXCR4) were constructed (Hanbio, Shanghai, China) to up-regulate the expression of CXCR4 *in vivo*, and empty vector served as negative control (AAV-NC). Briefly, intraperitoneal anesthesia was administered by pentobarbital sodium (50 mg/ kg), and mice were positioned in dorsal recumbency with their hindlimbs externally rotated. A skin incision was made over the femoral artery beginning at the inguinal ligament and continued caudally to the popliteal bifurcation. The unilateral femoral artery was isolated, doubly ligated using 7-0 Prolene suture, and transected, after separating the femoral artery from the femoral vein and nerve. Post ischemia, Exos (30 μg) suspended in a total volume of 100 μl PBS, AAV-CXCR4 (9–10 × 10^10^ viral particles per mouse), and AAV-NC were administered locally to ischemic gastrocnemius muscles (four injections per limb), respectively. After 24 h, the mice injected with DiI-labelled Exos were sacrificed, and the gastrocnemius muscles were taken for tracer experiment. Detection of hindlimb subcutaneous blood flow was performed using a laser Doppler imager (PERIMED PSI-ZR, Sweden). Blood flow in bilateral hindlimbs was measured at baseline and 0, 3, 7, 14 and 21 days after the operation. After that, mice were sacrificed, and the gastrocnemius muscles were taken for subsequent studies.

### Immunofluorescence Analysis

The thoracic aortas and gastrocnemius muscles were fixed in 4% paraformaldehyde, and then dehydrated and embedded in paraffin. Samples underwent dewaxing and antigen retrieval. The slides were blocked in 10% goat serum for 30 min at room temperature and then incubated with primary antibodies of CXCR4 (Abcam, MA, United States) and/or CD31 (Abcam, MA, United States) overnight. Slides were then incubated with Alexa Fluor^®^ 488 donkey anti-rabbit IgG (H + L) and/or Goat anti-Rabbit IgG (HRP). Slides were then washed and stained with DAPI (Solarbio, Beijing, China). The positive signals were detected with a fluorescence microscopy (Olympus, Tokyo, Japan).

### Statistical Analysis

Statistical analysis was conducted using the SPSS 20.0 software (SPSS Inc., Chicago, IL, United States), and the graphs were plotted by GraphPad Prism (San Diego, CA, United States). Data were presented as mean ± standard error of the mean (SEM). For continuous variables with normal distribution, the comparisons between two groups were performed with independent *t*-test, and comparisons among groups were performed with one-way analysis of variance (ANOVA) followed by least significant difference (LSD) test for pairwise comparisons. *P* < 0.05 was considered statistically significant.

## Results

### TMAO Stimulate Hepatocytes to Produce Exosomes

**FIGURE 1:**
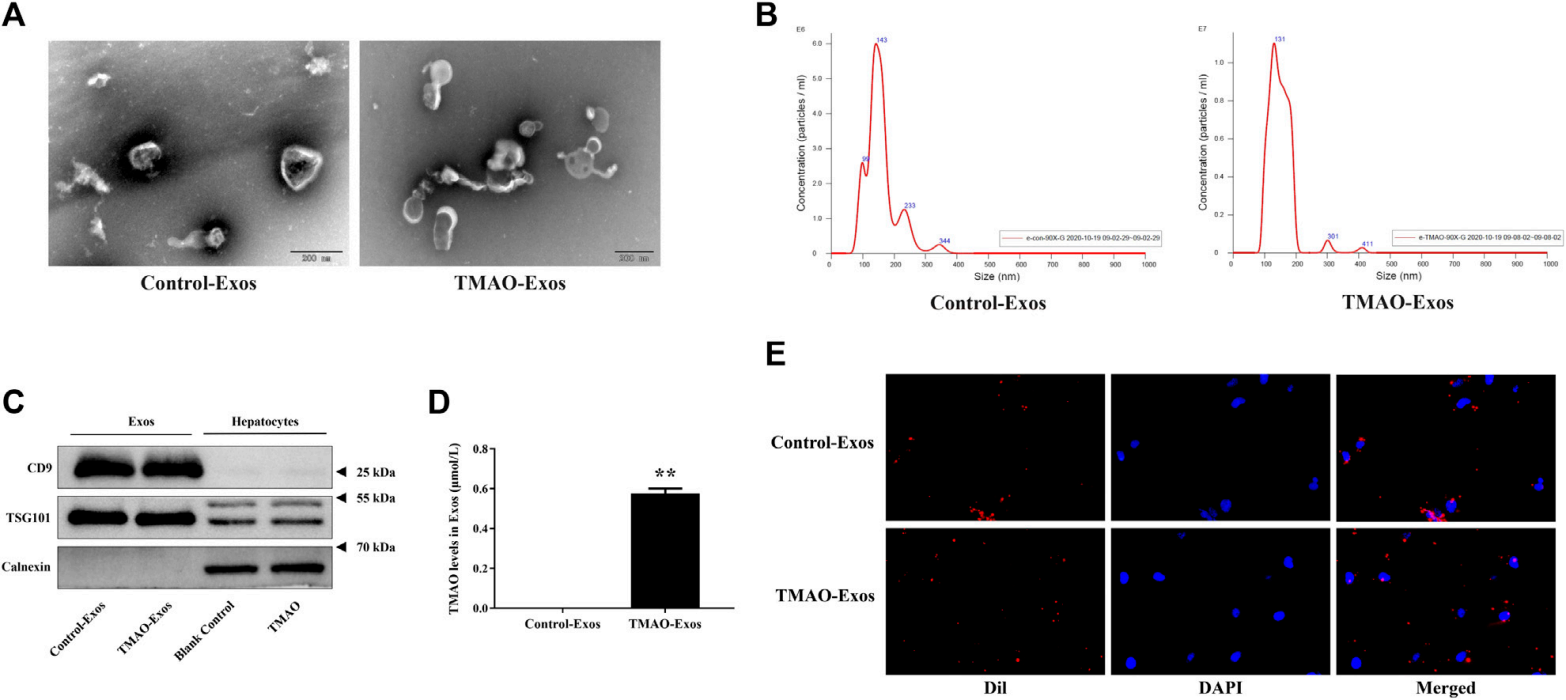
Isolation and characterization of exosomes from hepatocyte culture supernatant. **(A)** Nanovesicles with diameters around 100 nm were isolated and purified from the AML12 cell culture supernatant, which possessed the characteristic size range of exosomes (Exos) under electron microscopes. Bar: 200 nm. **(B)** The size distribution of the Exos showed no significant difference between trimethylamine-N-oxide (TMAO)-free group (Control-Exos) and TMAO-Exos. **(C)** Exosomal markers of CD9 and TSG101 were enriched in Exos groups, and the negative markers of calnexin were detected only in whole cell lysate. **(D)** TMAO was undetectable in Control-Exos, but a small quantity of TMAO remained in TMAO-Exos. Data were expressed as mean ± standard error of the mean (SEM). *n* = 3, independent *t*-test was performed for comparisons; **p < 0.01 versus Control-Exos. **(E)** Exosomes were labelled with DiI and co-cultured with human aortic endothelial cells (HAECs) for 24 h, and it was shown that DiI-labeled Exos could be taken up by cells (×400 magnification).

Nanovesicles with diameters around 100 nm were isolated and purified from the cell culture supernatant, and the size ranges were consistent with Exos under the electron microscope (Figure 1A). The size distribution of the Exos showed no significant difference between Control-Exos and TMAO-Exos (Figure 1B). Exosomal markers of CD9 and TSG101 were enriched in Exos groups, and the negative marker of calnexin was detected only in whole-cell lysate (Figure 1C). In addition, we tested the TMAO concentrations in Exos, and the results showed that TMAO was undetectable in Control-Exos, but a small quantity of TMAO remained in TMAO-Exos (Figure 1D). Furthermore, these DiI-labeled Exos could be taken up by HAECs (Figure 1E).

### TMAO-Exos Reduced CXCR4 Expression Independently of Alternative Splicing

**FIGURE 2:**
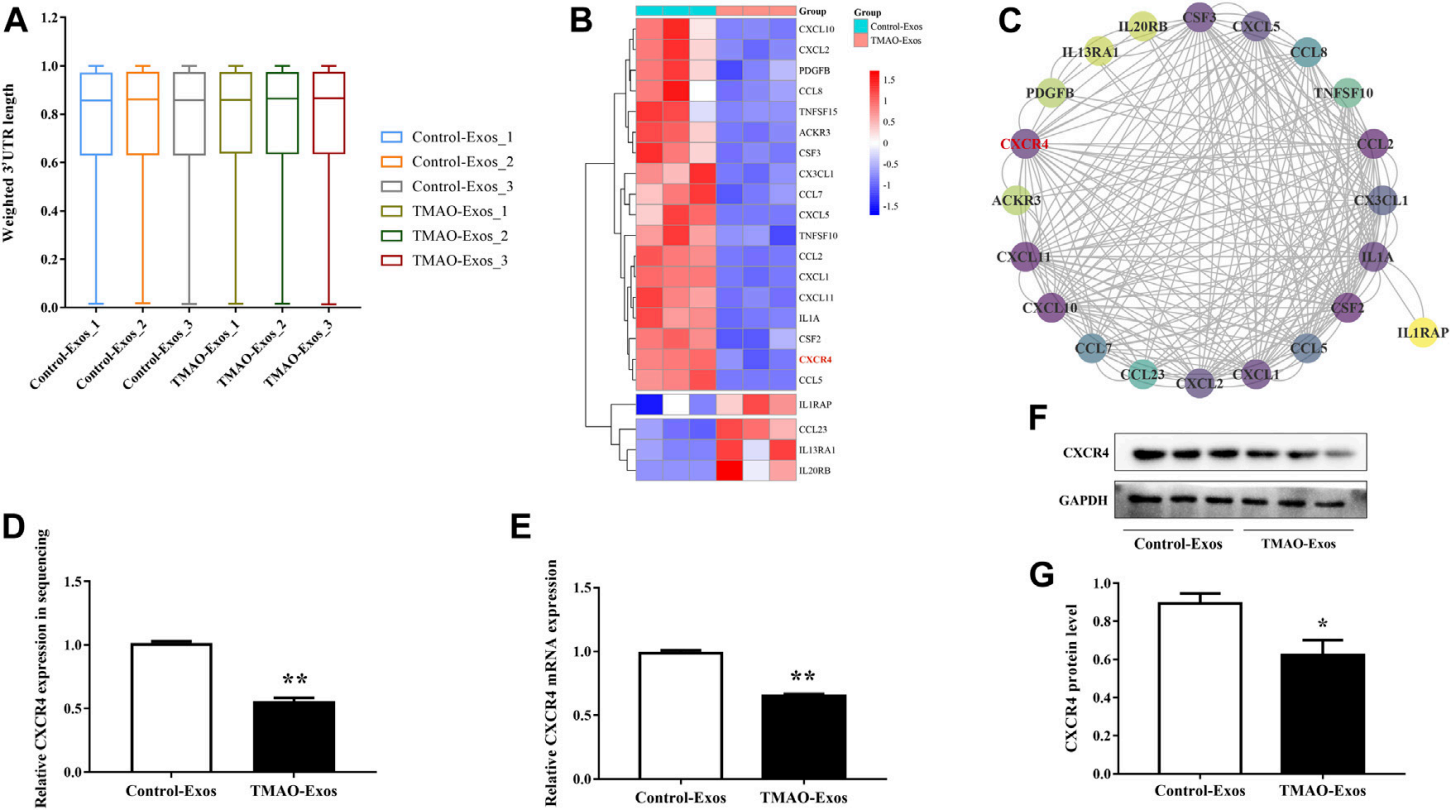
TMAO-Exos reduced C-X-C motif chemokine receptor 4 (CXCR4) expression independently of alternative splicing. **(A)** The tandem 3′-UTR lengths of genes in HAECs were not significantly regulated by Exos. **(B)** The genes in the “cytokine–cytokine receptor interaction” gene set were visualized on a heatmap. **(C)** CXCR4 was identified as a key gene according to the relative dark shade, with a degree of 30. Each node represents a gene, and the lines between nodes illustrate interactions between genes. The color of the nodes represents the degree of gene interaction. **(D−G)** The levels of CXCR4 in sequencing were further validated at the mRNA and protein levels. All data were expressed as mean ± SEM. *n* = 3, independent *t*-test was performed for comparisons; **p < 0.01, *p < 0.05 versus Control-Exos.

An APA-sequencing strategy was conducted to identify the APA profile in HAECs stimulated with Exos at the transcriptome level, and it was found that the tandem 3′-UTR lengths of genes were not significantly altered (Figure 2A). However, gene expression levels were changed remarkably in response to TMAO-Exos, and the differentially expressed genes were visualized on a heatmap (Supplementary Figure S1A). Compared to the Control-Exos group, a total of 293 genes changed significantly when exposed to TMAO-Exos, of which 84 were up-regulated and 209 were down-regulated. GO analysis showed that, in terms of molecular function, these genes were mainly enriched in signaling receptor binding, cytokine receptor binding, and cytokine activity (Supplementary Figure S1B), and KEGG analysis revealed that these genes were strongly enriched in the signal pathways of cytokine signaling in the immune system, ensemble of genes encoding extracellular matrix and extracellular matrix-associated proteins, interferon signaling, and cytokine–cytokine receptor interaction (Supplementary Figure S1C). Collectively, these analyses suggested that cytokine signaling and cytokine–cytokine receptor interaction were implicated. Therefore, the genes in the cytokine-related gene set, namely, “cytokine–cytokine receptor interaction,” were selected and visualized on a heatmap (Figure 2B), in which CXCR4 was identified as a hub gene, with a degree of 30 (Figure 2C). Furthermore, the levels of CXCR4 were validated to be notably decreased by TMAO-Exos (Figures 2D–G).

### TMAO-Exos Reduced CXCR4 Levels in the Aorta

**FIGURE 3:**
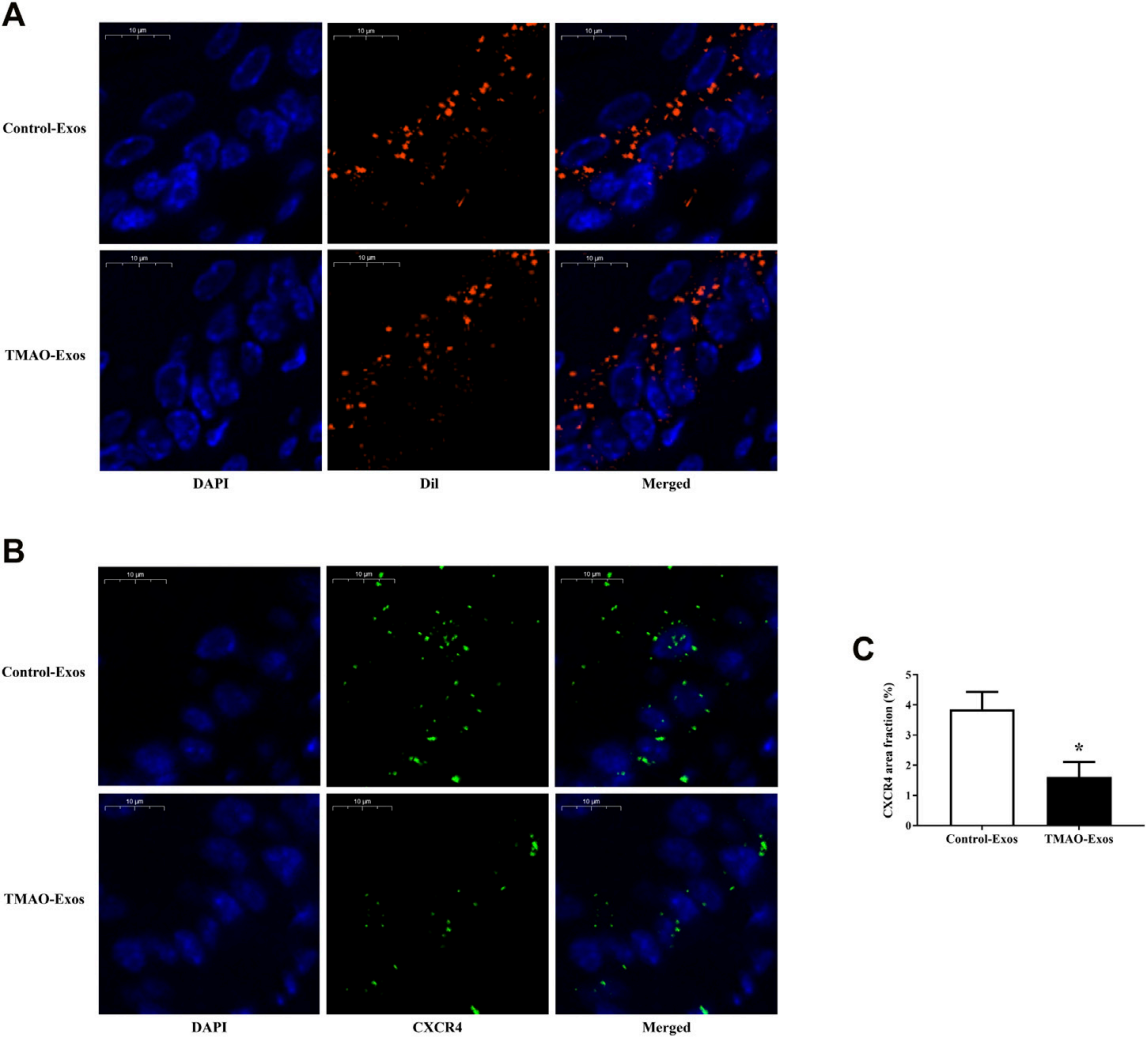
TMAO-Exos distributed and reduced CXCR4 levels in the aorta. **(A)** Representative photographs of the localization of DiI-labelled Exos in the aorta. Exos from both groups were mainly enriched in endothelial cells. Nuclei were stained with DAPI. Bar: 10 μm. **(B,C)** TMAO-Exos significantly reduced CXCR4 expressions. Nuclei were stained with DAPI. Bar: 10 μm. All data were expressed as mean ± SEM. *n* = 3, independent *t*-test was performed for comparisons; *p < 0.05 versus Control-Exos.

To clarify the relationships between Exos and endothelial cells *in vivo*, mice were intravenously injected with 30 μg of Exos or DiI-labelled Exos. After 24 h, the aortas were taken for detection of DiI signals and CXCR4 levels. The results showed that these Exos could be internalized by endothelial cells (Figure 3A), and furthermore, the CXCR4 expressions were significantly inhibited by TMAO-Exos (Figures 3B, C).

### TMAO-Exos Impaired Cell Migration and Tube Formation *via* Down-Regulation of CXCR4

**FIGURE 4:**
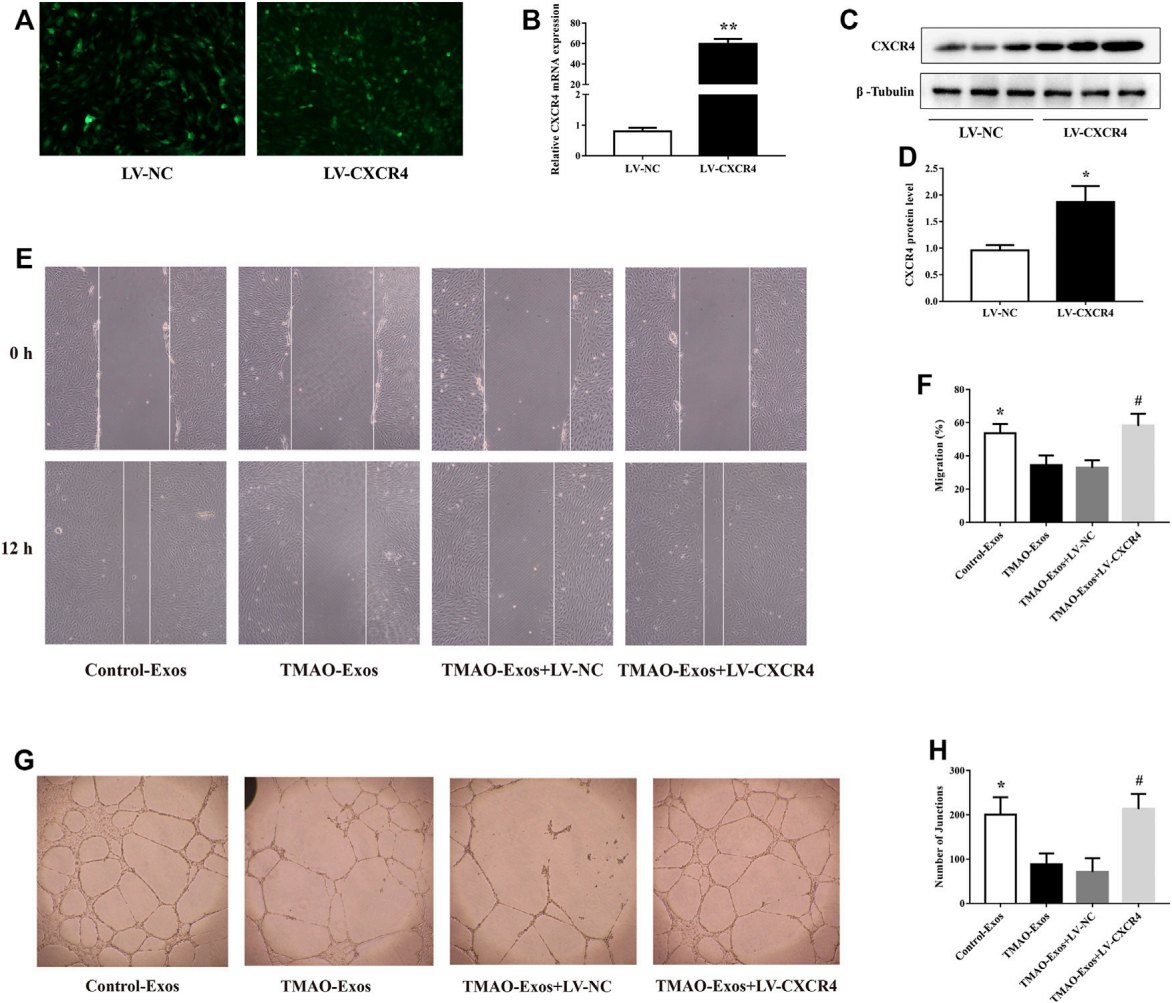
TMAO-Exos impaired cell migration and tube formation *via* down-regulation of CXCR4. **(A)** The GFP signals were detectable in HAECs transfected with LV-NC and LV-CXCR4 (×100 magnification). **(B–D)** Transfection of LV-CXCR4 significantly increased CXCR4 expression at the mRNA and protein levels. *n* = 3, independent *t*-test was performed for comparisons; **p < 0.01, *p < 0.05 versus the LV-NC group. **(E,F)** Cell migration (×100 magnification) and **(G,H)** tube formation (×40 magnification) were dramatically inhibited by TMAO-Exos, which could be rescued by overexpression of CXCR4. *n* = 3, one-way ANOVA followed by least significant difference (LSD) test for pairwise comparisons were applied; *p < 0.05, #*p* < 0.05 versus the TMAO-Exos group and the TMAO-Exos + LV-NC group. All data were expressed as mean ± SEM.

HAECs were transfected with LV-NC and LV-CXCR4, and after 24 h, the green fluorescence protein (GFP) signals were detectable in both groups (Figure 4A). Furthermore, transfection of LV-CXCR4, but not LV-NC, significantly increased the expression of CXCR4 (Figures 4B–D). In parallel, TMAO-Exos notably suppressed cell migration (Figures 4E, F) and tube formation (Figures 4G, H), which could be rescued by overexpression of CXCR4.

### TMAO-Exos Impeded Angiogenesis in HindLimb Ischemic Mice *via* Repressing CXCR4

**FIGURE 5:**
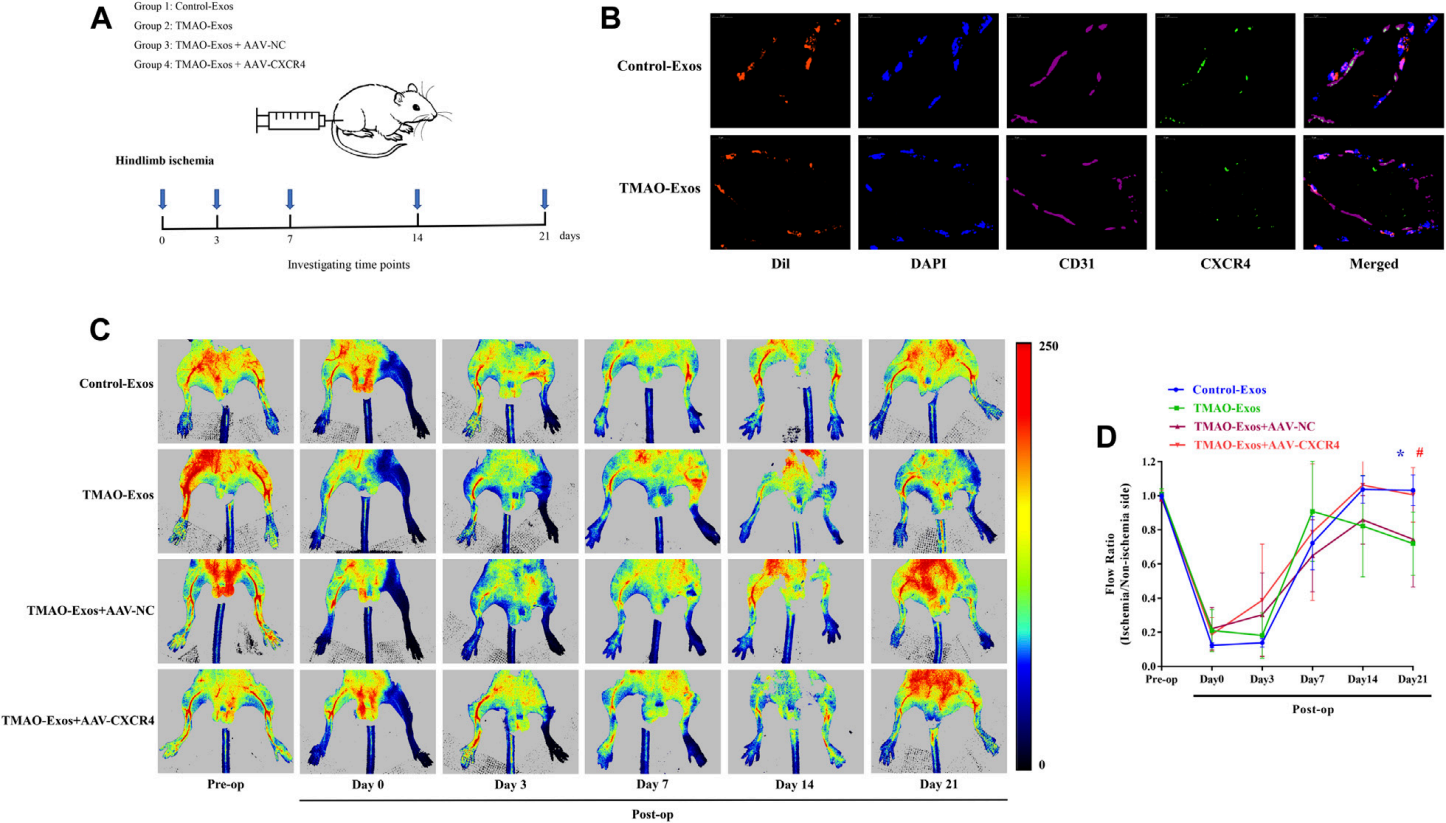
TMAO-Exos suppressed angiogenesis in hindlimb ischemic mice *via* repressing CXCR4. **(A)** The schematic representation for experimental workflow. **(B)** Representative photographs of the localization of DiI-labelled Exos in gastrocnemius muscles. Exos from both groups were mainly enriched in endothelial cells, where they were co-located with CXCR4. Nuclei were stained with DAPI. Bar: 10 μm. **(C,D)** TMAO-Exos significantly impeded revascularization at 21 days after ligation of the femoral artery. *n* = 6, one-way ANOVA followed by LSD test for pairwise comparisons were applied; *p < 0.05, #*p* < 0.05 versus the TMAO-Exos group and the TMAO-Exos + AAV-NC group. All data were expressed as mean ± SEM.

The *in vitro* results clearly showed that TMAO-Exos induced VEC dysfunction by reducing CXCR4 expression, and furthermore, studies by us and others have indicated that CXCR4 played an important role in promoting angiogenesis (Ziegler et al., 2016; Yu et al., 2019). Therefore, we next determined the effects of TMAO-Exos/CXCR4 pathway on revascularization in the mouse model of hindlimb ischemia, as shown in the schematic representation for experimental workflow (Figure 5A). To further reveal the relationships between Exos and endothelial cells *in vivo*, DiI-labelled Exos were intramuscularly injected. After 24 h, the gastrocnemius muscles were excised and stained with CD31 and CXCR4. As displayed, these Exos could be taken up by VECs, and both Exos and CXCR4 proteins had the same subcellular localizations in VECs (Figure 5B). Furthermore, TMAO-Exos significantly suppressed revascularization at 21 days after ligation of the femoral artery, as assessed by laser Doppler imaging (Figures 5C, D), which could be rescued by overexpression of CXCR4.

### TMAO-Exos Reduced CXCR4 Levels and Angiogenesis in Gastrocnemius Muscle

**FIGURE 6:**
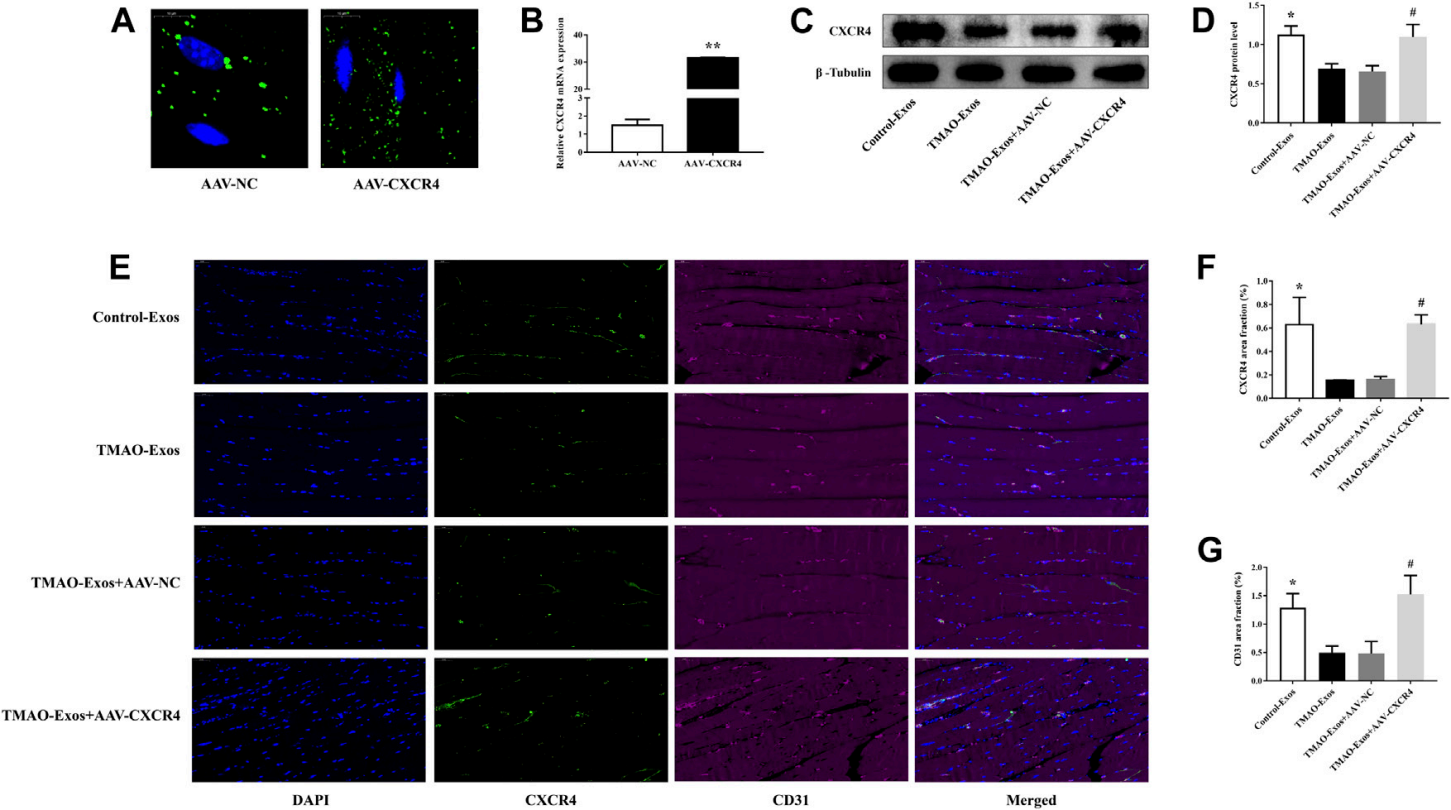
TMAO-Exos reduced the capillary density and CXCR4 expression *in vivo*. **(A)** The GFP signals of AAV-NC and AAV-CXCR4 were still detectable in gastrocnemius muscles after 21 days. Bar: 10 μm. **(B)** Transfection of AAV-CXCR4 efficiently increased the mRNA expression of CXCR4. *n* = 3, independent *t*-test was performed for comparisons; **p < 0.01. The CXCR4 expressions detected by western blot **(C,D)** and immunofluorescence analysis **(E,F)** were notably decreased by TMAO-Exos and restored by overexpression of CXCR4. *n* = 6 for western blot, *n* = 3 for immunofluorescence analysis, one-way ANOVA followed by LSD test for pairwise comparisons were applied; *p < 0.05, #*p* < 0.05 versus the TMAO-Exos group and the TMAO-Exos + AAV-NC group. Bar: 50 μm. **(E,G)** The capillary densities (indicated by CD31^+^ cells) were significantly down-regulated but rescued by amplifying the expression of CXCR4. *n* = 3, one-way ANOVA followed by LSD test for pairwise comparisons were applied; *p < 0.05, #*p* < 0.05 versus the TMAO-Exos group and the TMAO-Exos + AAV-NC group. Bar: 50 μm. All data were expressed as mean ± SEM.

After 21 days, the GFP signals of AAV-NC and AAV-CXCR4 were still detectable (Figure 6A), which contributed to a substantial increase of CXCR4 levels (Figure 6B). In addition, CXCR4 expressions determined by western blot (Figures 6C, D) and immunofluorescence analysis (Figures 6E, F) were remarkably decreased by TMAO-Exos and restored by AAV-CXCR4 transfection, which were consistent with the above-mentioned blood flow recovery (Figures 5C, D) and capillary density (Figure 6E, G). Taken together, these data indicated that TMAO-Exos significantly impeded revascularization *in vivo via* reducing CXCR4 expression.

## Discussion

In this work, we supplied further evidence that hepatocyte-derived Exos could be internalized by VECs, and furthermore, TMAO-activated Exos were able to suppress VEC function and angiogenesis by reducing CXCR4 expression, without altering the length of 3ʹ-UTR. These findings provided new ideas on an indirect link between TMAO and endothelial dysfunction.

An increasing body of evidence has suggested that TMAO plays an important part in vascular inflammation and endothelial dysfunction (Seldin et al., 2016; Li T. et al., 2017; Ke et al., 2018; Singh et al., 2019; Brunt et al., 2020; Chen et al., 2020; Liu and Dai, 2020). Furthermore, recent research showed that there existed a close relationship between TMAO and the liver (Chen et al., 2019; Coffey et al., 2019). TMAO was found to be able to target hepatocytes and exert its influence over metabolic syndrome (Chen et al., 2019), and data from animal models of atherosclerosis provided evidence that hepatic miR-146a-5p was aberrantly expressed, and its levels were associated with circulating TMAO (Coffey et al., 2019). Recently, we found that Exos isolated from TMAO-activated hepatocytes contained a distinctive profile of miRNAs, and furthermore, when taken up by VECs, TMAO-Exos, but not Control-Exos, notably promoted inflammation and damaged VECs and endothelium-dependent vasodilation (Liu et al., 2021b).

In recent years, Exos, including those from hepatocytes, have emerged as important players in regulating inflammation and vascular endothelial function (Hirsova et al., 2016; Zheng et al., 2017; Jiang et al., 2020; Xia et al., 2020). Besides, in high-fat diet-fed mice, the liver was deemed to be the candidate organ for the elevation of circulating arginase-1 levels by secreting Exos enriched in arginase-1. Enhanced arginase activity leads to reduced production of nitric oxide (Ogino et al., 2021). In the present study, we offered further evidence that Exos released from TMAO-activated hepatocytes were quite different from that in the TMAO-free group. Specifically, TMAO-Exos contributed to reduced cell migration and tube formation and impaired angiogenesis *in vivo*, at least in part, by decreasing CXCR4 expression. Although previous research has indicated that Exos could control alternative splicing (Zhang et al., 2015), TMAO-Exos failed to affect the splicing events in our study, which suggested that TMAO-Exos may regulate CXCR4 expression *via* an APA-independent manner. In addition, we found that there existed a small quantity of TMAO in TMAO-Exos; however, the average concentration was very low, especially given the dilution in treatment, and thus, it looked unlikely to exert an influence on VECs.

CXCR4 is constitutively expressed in numerous tissues, including endothelial and hematopoietic cells (Petit et al., 2007). As one of the most important receptors for stromal cell-derived factor 1, CXCR4 has been known to play a central role in angiogenesis, endothelial function, and atherosclerosis (Petit et al., 2007; Zernecke et al., 2008; Chen et al., 2010; Noels et al., 2014; Ziegler et al., 2016; Doring et al., 2017). Our previous work demonstrated that overexpression of CXCR4 in endothelial progenitor cells improved *in vitro* function and *in vivo* reendothelialization capacity through enhancing Janus kinase-2 activity (Chen et al., 2010). Furthermore, CXCR4 was involved in mediating the function of Exos. Ciullo et al. (2019) demonstrated that overexpression of CXCR4 in Exos, which derived from cardiac-resident progenitor cells, could significantly decrease infarct size and improve left ventricle ejection fraction. Another research showed that Exos were efficient in transferring myocardial miRNAs to the bone marrow, thus down-regulating CXCR4 expression and promoting the mobilization of progenitor cells (Cheng et al., 2019). Accordingly, it is inferred that the inhibiting effect of TMAO-Exos on CXCR4 may be related to the abnormally enriched miRNAs. In our previous study, miR-302d-3p, miR-302b-3p, and miR-302a-3p were found to be enriched in TMAO-Exos (Liu et al., 2021b). It is worth noting that miR-302d-3p, miR-302b-3p, and miR-302a-3p are the members of the miR-302/367 cluster, which is highly conserved and plays a pivotal role in cell proliferation, differentiation, and reprogramming (Guo et al., 2020). Actually, studies have revealed that miR-302/367 cluster can inhibit CXCR4 expression (Fareh et al., 2012; Fareh et al., 2017). Therefore, we speculate that TMAO-Exos may reduce CXCR4 by transferring miR-302d-3p, miR-302b-3p, and miR-302a-3p to the recipient cells. Similar findings were reported in a previous research, in which cardiomyocytes were found to exert inhibitory effects on angiogenesis in type 2 diabetic rats by transferring exosomal miR-320 into cardiac endothelial cells and repressing the target genes of IGF-1, Hsp20, and Ets2 (Wang et al., 2014).

The present study has its strength and limitations. We offered further evidence that hepatocyte-derived Exos could mediate the adverse effects of TMAO upon endothelial function, which may extend our current knowledge on the biologic behavior of TMAO. However, the exact way how TMAO works and how hepatocytes secrete Exos still remains elusive. It was found that the release of Exos from hepatocytes was differentially regulated by CXCR1 and CXCR2 (Nojima et al., 2016). In addition, the exact mechanism by which TMAO-Exos reduced CXCR4 expression needs to be further elucidated in a follow-up study.

## Conclusion

In short, our findings revealed that Exos secreted from TMAO-activated hepatocytes were quite different from those in the TMAO-free group, and after taken up by VECs, TMAO-Exos, but not Control-Exos, significantly reduced cell migration and tube formation *in vitro* and impaired perfusion recovery and angiogenesis after hindlimb ischemia, by down-regulating the expression of CXCR4. These findings provided new ideas on an indirect mechanism by which TMAO regulates endothelial function, which may offer a novel target for restraining the detrimental effects imposed by TMAO.

## Data Availability

According to national legislation/guidelines, specifically the Administrative Regulations of the People’s Republic of China on Human Genetic Resources (http://www.gov.cn/zhengce/content/2019-06/10/content_5398829.htm, http://english.www.gov.cn/policies/latest_releases/2019/06/10/content_281476708945462.htm), no additional raw data is available at this time. Data of this project can be accessed after an approval application to the China National Genebank (CNGB, https://db.cngb.org/cnsa/). Please refer to https://db.cngb.org/, or email: CNGBdb@cngb.org for detailed application guidance. The accession code HRA001467 should be included in the application.

## Glossary

TMAO: trimethylamine-N-oxide
Exos: exosomes
TMAO-Exos: exosomes isolated from TMAO-treated group
Control-Exos: exosomes isolated from TMAO-free group
VECs: vascular endothelial cells
APA: alternative polyadenylation
3ʹ UTR: 3ʹ untranslated region
CXCR4: C-X-C motif chemokine receptor 4
AML12: alpha mouse liver 12
DMEM/F12: Dulbecco’s Modified Eagle Medium/Nutrient Mixture F-12
PBS: phosphate-buffered saline
BCA: bicinchoninic acid
TSG101: tumor susceptibility 101
DiI: 1,1′-dioctadecyl-3,3,3′3′-tetramethylindocarbocyanine perchlorate
LC-MS/MS: liquid chromatography-tandem mass spectrometry
HPLC: high performance liquid chromatography
ESI: electrospray ionization
HAECs: human aortic endothelial cells
ECM: endothelial cell medium
LV-CXCR4: lentiviral vector carrying CXCR4 gene
LV-NC: empty lentiviral vector
DAVID: Database for Annotation, Visualization, and Integrated Discovery
GO: Gene Ontology
KEGG: Kyoto Encyclopedia of Genes and Genomes
PPI: protein–protein interactions
SDS-PAGE: sodium dodecyl sulfate polyacrylamide gel electrophoresis
GAPDH: glyceraldehyde phosphate dehydrogenase
HRP: horseradish peroxidase
qPCR: quantitative polymerase chain reaction
cDNA: complementary deoxyribonucleic acid
AVV-CXCR4: adeno-associated virus vector carrying CXCR4 gene
AAV-NC: empty adeno-associated virus vector
DAPI: 4′,6-diamidino-2-phenylindole
SEM: standard error of the mean
ANOVA: one-way analysis of variance
IGF-1: insulin like growth factor 1
Hsp20: heat-shock protein 20
Ets2: ETS proto-oncogene 2, transcription factor
CXCR1: C-X-C motif chemokine receptor 1
CXCR2: C-X-C motif chemokine receptor 2.
