# Supplementary figures and images for: TMAO-Activated Hepatocyte-Derived Exosomes Impair Angiogenesis via Repressing CXCR4
Source: Front Cell Dev Biol. 2022 Jan 31;9:804049. doi: 10.3389/fcell.2021.804049 (PMC8841965; doi:10.3389/fcell.2021.804049)

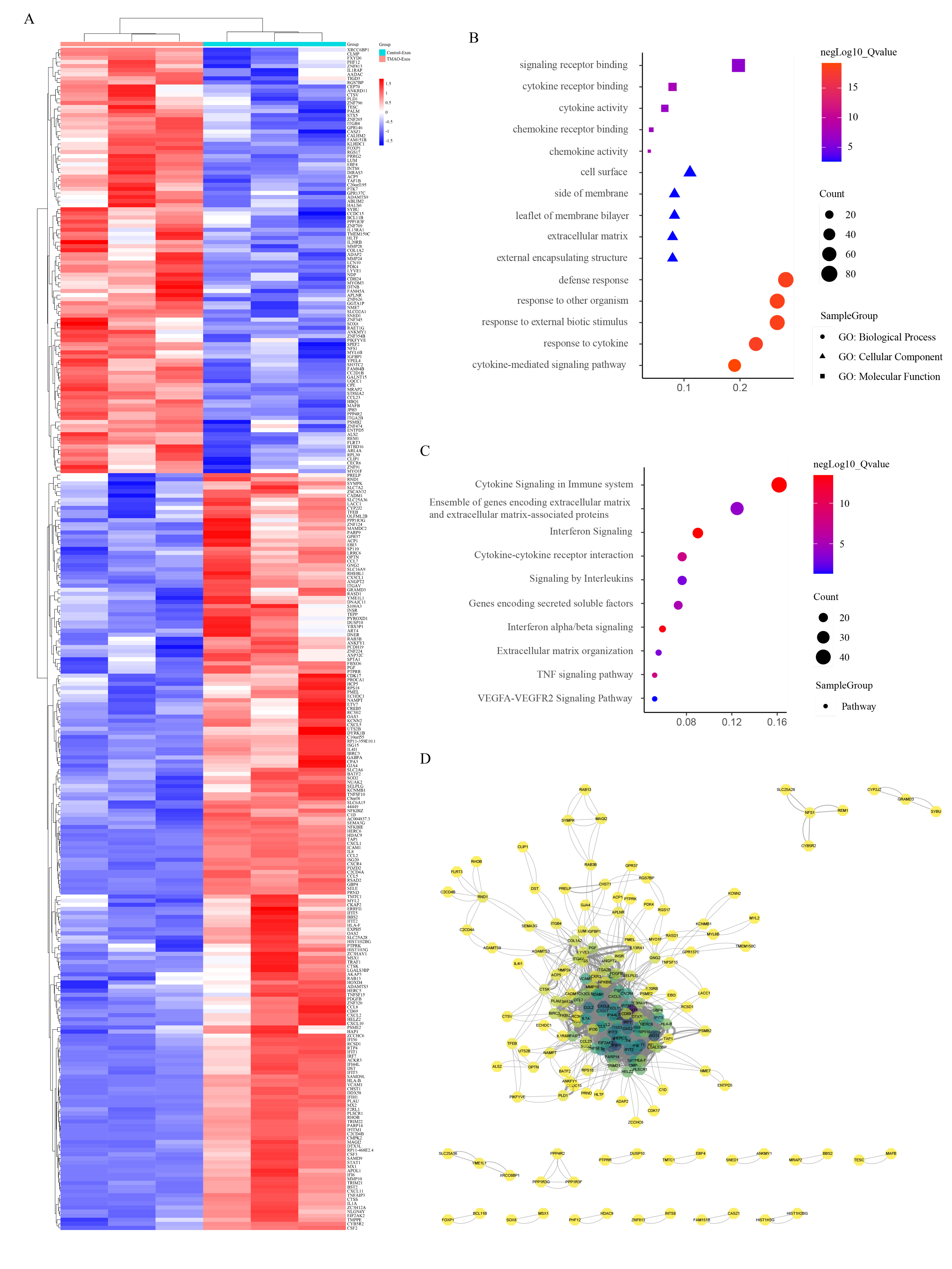

Supplement: Supplementary file 1 [file Image1.JPEG]
